# Overcoming immunotherapy resistance in triple-negative breast cancer: a critical review of mast cell plasticity, metabolic reprogramming, and organoid models

**DOI:** 10.3389/fimmu.2026.1774946

**Published:** 2026-05-25

**Authors:** Kunyuan He, Shaofeng Yang, Ke Zhang, Ling Yang, Fei Song

**Affiliations:** 1 Department of Thyroid Breast Surgery, The Affiliated Hospital of Inner Mongolia Medical University, Hohhot, Inner Mongolia Autonomous Region, China; 2 The Central Laboratory, The Affiliated Hospital of Inner Mongolia Medical University, Hohhot, Inner Mongolia Autonomous Region, China

**Keywords:** immunotherapy resistance, mast cell, metabolic reprogramming, organoid, triple-negative breast cancer

## Abstract

Immunotherapy resistance in triple-negative breast cancer (TNBC) remains a critical clinical challenge. This review synthesizes and critically evaluates current evidence on two interconnected mechanisms that have emerged from recent research: the functional plasticity of mast cells (MCs) and metabolic reprogramming. Far from being mere allergic effectors, MCs exhibit high phenotypic diversity, with antigen-presenting MCs (apMCs) representing a unique subset identified in recent studies capable of priming anti-tumor T cell responses. A landmark phase 2 trial has demonstrated that modulation of apMCs can enhance responses to anti-PD-1 therapy in patients with ICI-refractory TNBC. However, within the TNBC TIME, metabolic dysregulation—characterized by glycolytic flux, lactate accumulation, and lipid alterations—skews MCs toward immunosuppressive phenotypes and suppresses apMC function. Published studies have documented that this bidirectional crosstalk forms a vicious cycle that sustains immune evasion and limits the efficacy of immune checkpoint inhibitors. To decipher this complexity, patient-derived organoid (PDO) models co-cultured with autologous immune cells have emerged as a validated platform that preserves tumor heterogeneity and enables real-time dissection of metabolism-immune circuits. This review systematically synthesizes current knowledge on the biological basis of MC plasticity, the metabolite-driven regulation of their function, and the utility of organoid-based systems for mechanistic discovery and drug screening. We critically evaluate emerging multidimensional therapeutic strategies, including pharmacological reprogramming of apMCs, metabolic normalization, and engineered cell therapies, and identify key knowledge gaps that must be addressed to translate these insights into clinical practice. By integrating advances in immuno-oncology, cancer metabolism, and bioengineering, this review provides a framework for translating current insights into clinically actionable strategies to overcome immunotherapy resistance in TNBC.

## 1 Introduction

Triple-negative breast cancer (TNBC) remains a formidable clinical challenge due to its aggressive nature, lack of targetable receptors, and poor prognosis (1). Although the advent of immune checkpoint inhibitors (ICIs), particularly anti-PD-1/PD-L1 agents, has revolutionized the treatment landscape for advanced TNBC (2, 3), their clinical benefit is limited by pervasive primary and acquired resistance (4, 5). Data from phase III trials show that objective response rate (ORR) to ICI-chemotherapy combinations in metastatic settings remains around 50-60% even in PD-L1-positive cohorts, and most patients eventually progress (6, 7). Mechanistic studies have demonstrated that this resistance stems not from a singular defect but from a complex, adaptive ecosystem-the tumor immune microenvironment (TIME) (5). This microenvironment is characterized by dysfunctional immune cells, an immunosuppressive network involving regulatory T cells (Tregs), tumor-associated macrophages (TAMs), and myeloid-derived suppressor cells (MDSCs), as well as a metabolically hostile landscape (8, 9).

Traditional research models, including two-dimensional cell lines and animal models, fall short in recapitulating the human-specific heterogeneity and dynamic multicellular interactions within this ecosystem. This review synthesizes current evidence on two interconnected mechanisms that sustain the immunosuppressive TNBC-TIME. Recent studies have highlighted two interconnected themes that contribute to immunosuppression in the TNBC TIME: the functional plasticity of mast cells and metabolic reprogramming. The functional plasticity of immune cells, with a spotlight on mast cells (MCs) (10). Studies have shown that far beyond their classical role as allergic effector cells, MCs exhibit a remarkable spectrum of functions within tumors (11, 12). Recent evidence highlights a unique subset with antigen-presenting capabilities—antigen-presenting mast cells (apMCs)—which can be pivotal for anti-tumor immunity, yet their default state in TNBC often leans towards pro-angiogenic and immunosuppressive phenotypes (13, 14). The second pillar is tumor cell-intrinsic and microenvironmental metabolic reprogramming. Multiple studies have documented that TNBC cells exhibit a profound “Warburg effect,” glutamine addiction, and dysregulated lipid metabolism (15), which not only fuel tumor growth but also create a metabolically suppressive milieu that directly inhibits effector immune cell function and may skew MC plasticity (16, 17).

**Figure 1:**
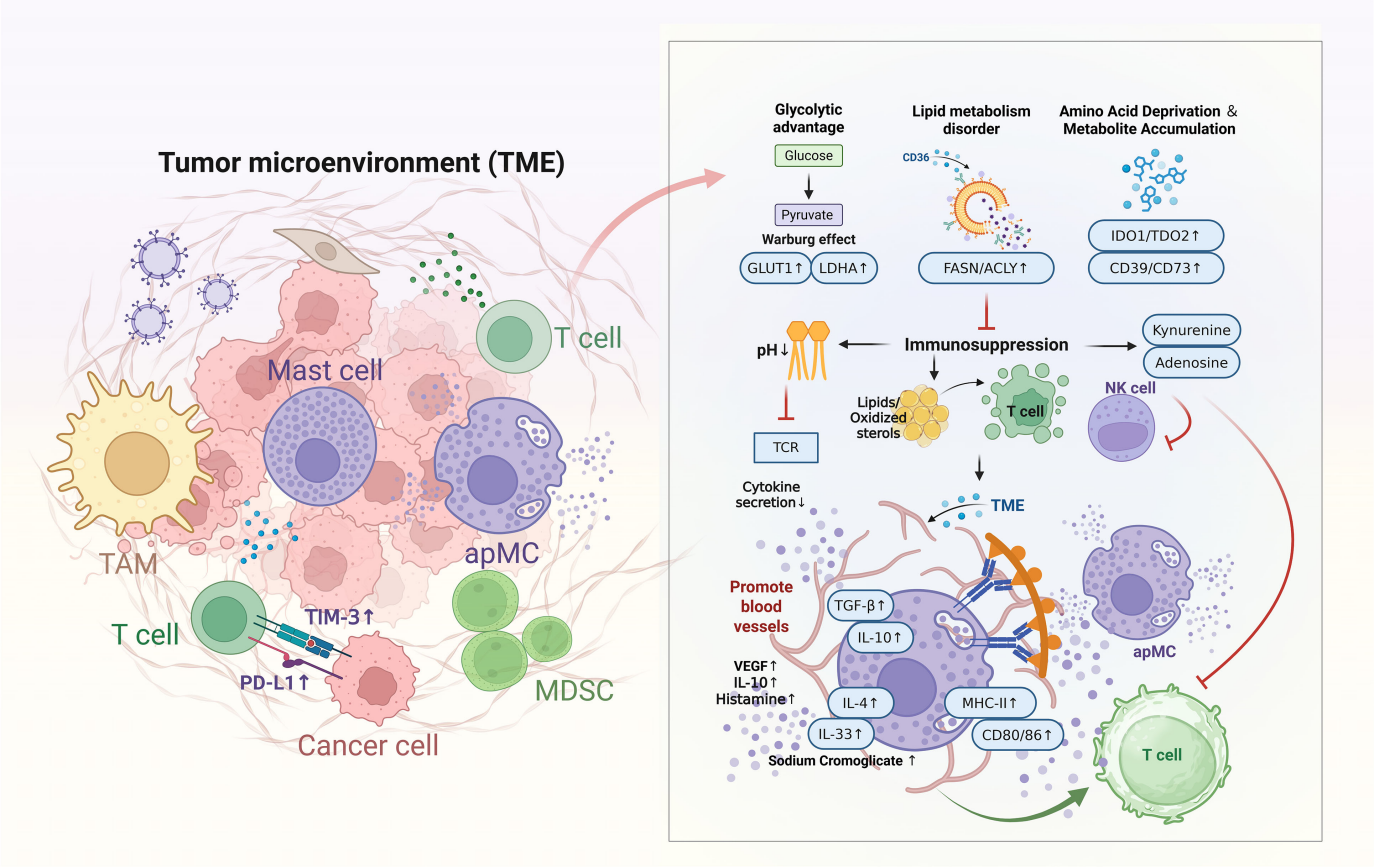
This schematic illustrates the two interconnected pillars that lead to resistance to immune checkpoint inhibitors (ICI). Metabolic reprogramming: tumor cells collectively shape a “metabolic checkpoint” that suppresses immune cell function by enhancing glycolysis (Warburg effect, producing lactate), dysregulating lipid metabolism (e.g., uptake via CD36, synthesis via FASN/ACLY), and depleting amino acids/accumulating toxic metabolites (e.g., through IDO1/TDO2, CD39/CD73 pathways). Immune ecological imbalance: focusing on the functional plasticity of mast cells (MC), it shows their fate decisions within the TME. Under inhibitory signals (e.g., TGF-β, IL-10), MCs differentiate into pro-angiogenic/immunosuppressive phenotypes, secreting VEGF, histamine, and other factors to promote immune evasion. In contrast, under intervening signals (e.g., sodium cromoglicate, IL-4, IL-33), MCs can be reprogrammed into antigen-presenting mast cells (apMCs), with high expression of MHC-II and CD80/86, thereby activating anti-tumor T cell responses.

Crucially, these pillars do not operate in isolation. Published evidence indicates that a vicious cycle exists where metabolic reprogramming drives immunosuppressive phenotypes, which in turn reinforce metabolic dysfunction. Deciphering this synergistic interplay is essential for developing effective interventions. To this end, patient-derived organoid (PDO) models have emerged as a validated platform. PDOs can preserve the genetic, phenotypic, and architectural complexity of the parent tumor *in vitro* (18). When combined with autologous immune cells—including MCs—in advanced co-culture systems, they enable the reconstruction of a personalized “mini-TIME” (19–21). This allows for the real-time, dynamic, and controlled investigation of the causal relationships between tumor metabolism, immune cell phenotype, and therapy response (**Figure 1**).

The primary objective of this review is to critically evaluate and synthesize current evidence on how mast cell plasticity and metabolic reprogramming contribute to immunotherapy resistance in TNBC. Furthermore, we will demonstrate how advanced organoid models serve as a validated tool for dissecting this complex crosstalk and for screening combination strategies aimed at reprogramming apMCs and normalizing the metabolic microenvironment to restore anti-tumor immunity.

## 2 Overview of TNBC immunological characteristics and current immunotherapy challenges

### 2.1 Immunological characteristics of TNBC

TNBC, as a highly heterogeneous breast cancer subtype, presents unique complexity in its immunological features. Large-cohort studies have established that compared to other breast cancer subtypes, TNBC typically has a higher tumor mutational burden (TMB) and richer tumor-infiltrating lymphocytes (TILs), providing a biological basis for it being a potential target for immunotherapy (22). Single-cell RNA sequencing analysis has revealed a unique immunosuppressive microenvironment in TNBC, characterized by a higher proportion of Tregs and exhausted CD8+ T cells, accompanied by abundant plasma cells, collectively shaping its immunological characteristics (23). However, this immune infiltration is not always associated with good prognosis or treatment response. Studies have shown that in triple-negative inflammatory breast cancer, despite the presence of immunosuppressive tumor-infiltrating immune components, the tumor mutational burden is lower than in non-inflammatory TNBC and is associated with poor response to neoadjuvant chemotherapy, highlighting the complexity of TNBC immune features (24).

Current State: It is now established that TIL density is prognostic in TNBC, but the functional state and spatial organization of immune infiltrates are critical determinants of outcome.

Knowledge Gaps: The dynamic evolution of immune infiltrates during treatment and the mechanisms by which tertiary lymphoid structures enhance anti-tumor immunity remain incompletely understood.

Future Directions: Longitudinal sampling of tumors before, during, and after therapy, combined with spatial transcriptomics, could reveal how immune architecture evolves and identify optimal windows for intervention.

### 2.2 Current immunotherapy challenges of TNBC

The application of ICI in TNBC has ushered in an era marked by both promise and disappointment. Phase III trials such as IMpassion130 and KEYNOTE-355 established atezolizumab or pembrolizumab combined with chemotherapy as the first-line standard for PD-L1-positive (defined by SP142 or CPS ≥ 10) metastatic TNBC, extending median progression-free survival (PFS) by approximately 2–3 months. with a trend towards overall survival (OS) benefit also observed (7, 25). Yet this “success” masks a stark reality: published efficacy data show that even within PD-L1-positive cohorts, the ORR for combination therapy hovers around 50-60%, indicating nearly half of patients exhibit primary resistance from the outset (6). For PD-L1-negative patients, the benefit from immunotherapy is negligible, highlighting the limitations of relying on a single biomarker. PD-L1 expression itself exhibits heterogeneity (tumor cells vs. immune cells), spatial heterogeneity (primary tumor vs. metastatic sites), and dynamic changes, all of which constrain its predictive value (26, 27).

Acquired resistance presents an even greater challenge. The KEYNOTE-522 study demonstrated that pembrolizumab combined with chemotherapy significantly improved pathological complete response (pCR) rates in the neoadjuvant setting (28). Nevertheless, 40-50% of patients still failed to achieve pCR, and a proportion of those who did experience long-term recurrence, suggesting that the initial effective immune response was undermined by tumor adaptive mechanisms (29).This adaptive mechanism is not a single pathway but a multi-layered, dynamically evolving ecosystem. Under immune editing pressure, tumors may achieve “immune invisibility” by downregulating key components of HLA-I assembly (such as β2-microglobulin) or by losing highly immunogenic neoantigens (e.g., those arising from frameshift mutations) through genomic instability (30, 31). Concurrently, immunosuppressive networks within the tumor microenvironment (TME) undergo systematic reinforcement: CD8^+^ T cells exhibiting exhausted phenotypes proliferate, co-expressing multiple inhibitory receptors (e.g., PD-1, TIM-3, LAG-3, TIGIT) with an epigenetically fixed state resistant to ICI reversal (32); MDSCs and M2 TAMs employ mechanisms such as arginase-1, nitric oxide synthase, and indoleamine 2,3-dioxygenase (IDO), depriving T cells of essential amino acids and generating inhibitory metabolites, whilst simultaneously secreting cytokines such as TGF-β and IL-10 (33); Tregs directly degrade CD80/CD86 molecules on antigen-presenting cells (APCs) via CTLA-4-mediated “trans-endocytosis”, or generate adenosine through high expression of CD39/CD73, thereby weakening co-stimulatory signals and suppressing effector T cell function (32–35).

Existing combination strategies attempt a multi-pronged approach but often encounter the “addition dilemma”. While ICI combined with chemotherapy can increase tumor antigen release and dendritic cell activation by inducing immunogenic cell death (ICD, e.g., anthracyclines, oxaliplatin), chemotherapeutic agents (particularly taxanes, gemcitabine) also exert cytotoxic effects on proliferating lymphocytes, potentially offsetting some immunological benefits. Optimal sequencing and dosing remain to be explored (36). ICI combination with anti-angiogenic agents (e.g., bevacizumab) aims to reverse tumor vascular abnormalities and hypoxia, thereby enhancing immune cell infiltration. However, the window for vascular normalization is transient and challenging to time accurately, potentially exacerbating toxicities such as hemorrhage and hypertension (37). The combination of ICIs with PARP inhibitors shows promise in BRCA-mutated TNBC, though its synergistic mechanisms (e.g., enhancing genomic instability, activating the cGAS-STING pathway) yield limited efficacy in non-BRCA-mutated tumors (38). While these regimens may enhance limited therapeutic responses, they are often accompanied by additive toxicities-such as severe immune-related pneumonia, colitis, myocarditis, and myelosuppression-which constrain their widespread application (39). Consequently, the next phase of breakthroughs must be grounded in a more refined understanding of mechanisms, shifting from a “carpet bombing” approach to “precision surgery” targeting key pivotal nodes, alongside the development of tools capable of predicting and monitoring the dynamics of drug resistance.

Current State: Multiple resistance mechanisms have been identified through analysis of clinical trial samples, confirming the multifaceted nature of ICI resistance.

Knowledge Gaps: The relative contribution of each mechanism varies among patients, and predictive biomarkers for specific resistance pathways are lacking.

Future Directions: Developing functional assays (e.g., using organoid co-culture systems) to prospectively identify dominant resistance mechanisms in individual patients could enable personalized combination strategies.

## 3 Mast cells in the TNBC immune microenvironment—from basic biology to therapeutic target

### 3.1 Biological characteristics and clinical relevance of mast cells in TNBC

Mast cells are tissue-resident innate immune cells of myeloid origin, traditionally recognized for their role in IgE-mediated allergic reactions. Their cytoplasm is rich in pre-synthesized granules containing mediators such as histamine, tryptase, and chymase (40, 41). Basic research has expanded our understanding of mast cell function. Upon activation via the high-affinity IgE receptor (FcϵRI), they rapidly degranulate, releasing mediators that can cause acute reactions like vasodilation and smooth muscle contraction (42). However, recent research has greatly expanded our understanding of mast cell function. Beyond the classical FcϵRI pathway, mast cells also express various other receptors, such as the Mas-related G protein-coupled receptor X2 (MRGPRX2) (43). Additionally, mast cells can regulate macrophage differentiation and polarization by releasing pre-stored mediators like colony-stimulating factor 1 (CSF1) (44). These findings collectively reveal the diversity and complexity of mast cell functions, far beyond their traditional positioning as mere “allergic effector cells.” (**Table 1**).

**Table 1:** Summary of research on the role of mast cells in tumors.

Disease	Mast cell function	Research model (*in vitro*/*in vivo*/clinical study)	Reference
Triple-negative breast cancer	Enhances response to PD-1 blockade immunotherapy	*In vitro*/clinical study (NCT05076682)	(14)
Triple-negative breast cancer	Promoting the recruitment and activation of T cells and B cells and coordinating anti-tumor immune responses	*In vitro*/*in vivo*	(13)
Breast cancer	Exerting anti-apoptotic effects	*In vitro*/*in vivo*	(45)
Hepatocellular carcinoma	Intratumoral infiltration is associated with good prognosis	*In vitro*	(46)
Adrenocortical carcinoma	Intratumoral infiltration is associated with good prognosis	*In vitro*	(47)
Pancreatic ductal adenocarcinoma	High-density infiltration is associated with aggressive phenotype, immunosuppression, and poor prognosis	*In vitro*/*in vivo*	(48)
High-grade serous ovarian cancer	High-density infiltration is associated with immunosuppressive microenvironment and poor prognosis	*In vitro*	(49)
Colorectal cancer	Pro-inflammatory phenotype, releases IL-6 and TNF-α, supporting tumor progression	*In vitro*/*in vivo*	(50)
Cutaneous squamous cell carcinoma	Induces degranulation and IL-17A secretion, promoting tumor progression	*In vitro*/*in vivo*	(51)

In tumor biology, the relationship between mast cell infiltration and patient prognosis exhibits a high degree of “paradox” and context-dependency, profoundly reflecting their functional heterogeneity and plasticity. Studies have shown that in certain tumor types, such as hepatocellular carcinoma (46) and adrenocortical carcinoma (47), intra-tumoral mast cell infiltration is associated with better prognosis and anti-tumor immune features. However, in many other cancers, including pancreatic ductal adenocarcinoma (48), high-grade serous ovarian cancer (49), a high density of tumor-associated mast cells is significantly correlated with more aggressive disease phenotypes, immunosuppressive tumor microenvironments, and worse clinical outcomes. This seemingly contradictory phenomenon suggests that mast cells do not simply exert pro- or anti-tumor effects; rather, their function is highly dependent on local microenvironmental signals and their own activation state. Therefore, the role of mast cells in tumors is highly contextual, and their functional “duality” is central to understanding their clinical relevance.

The application of new technologies like single-cell RNA sequencing provides unprecedented perspectives for parsing the high heterogeneity of mast cells in the tumor microenvironment. These studies reveal that mast cells are not a homogeneous population within different tissues or even the same tumor but exist as subsets with different gene expression profiles and functional potentials (10). In triple-negative breast cancer, research has identified a mast cell subset with antigen-presenting capacity. This subset is associated with better response to PD-1 blockade immunotherapy and may effectively present and cross-present antigens, activating anti-tumor T cell immunity, likely located within tertiary lymphoid structures (14). Conversely, in other tumor models, certain mast cell subsets exhibit features promoting immunosuppression and chemotherapy resistance (45, 52). This heterogeneity at the subset level directly leads to their functional diversity. Dynamic analysis of mast cell-specific protease (e.g., tryptase, chymase A3) expression also supports this view, showing significant spatial heterogeneity in the transcriptional and translational upregulation of these proteases in different pathological states like chronic lung diseases, correlating with immunopathological changes and functional decline (53). Therefore, defining functionally distinct mast cell subsets based on their molecular phenotype and spatial localization, rather than treating them as a monolithic entity, is crucial for accurately assessing their clinical significance in specific diseases like TNBC and developing precise intervention strategies.

Current State: Mast cell heterogeneity is well established, and a specific apMC subset has been identified in TNBC.

Knowledge Gaps: The origin, stability, and lineage relationships of different mast cell subsets in the TME remain unclear.

Future Directions: Lineage tracing studies and single-cell epigenetic profiling could clarify how mast cell subsets arise and whether they represent distinct lineages or plastic states.

### 3.2 Functional plasticity of mast cells: phenotypic spectrum and regulatory network

Multiple studies have demonstrated that mast cells, as tissue-resident immune cells, exhibit high plasticity rather than fixed function, able to polarize into phenotypes with different or even opposing functions according to microenvironmental signals (54). This plasticity allows mast cells to play complex roles in the tumor microenvironment, with their functional spectrum potentially shifting from the classic pro-angiogenic/immunosuppressive type to the emerging antigen-presenting type (apMC) (55). tumor-associated mast cells primarily present a connective tissue phenotype and releIn colorectal cancer models, ase high levels of interleukin-6 (IL-6) and tumor necrosis factor-alpha TNF-α, thereby maintaining a pro-inflammatory microenvironment that supports tumor progression. The polarization of this phenotype is closely related to persistent stimulation by high levels of stem cell factor (SCF) and IL-33 within the tumor (50). Furthermore, interactions between mast cells and neighboring immune cells like macrophages further shape their function. Mediators released by activated mast cells can induce epigenetic reprogramming in macrophages, endowing them with a polarization phenotype different from the classical M1/M2 paradigm, profoundly altering macrophage phagocytic function, cytokine production, and transcriptomic responses (56). This “peripheral immune education” through cell-cell contact or soluble mediators allows mast cells to adjust their function based on the local tissue environment, forming a complex regulatory network.

Key signaling pathways regulating mast cell plasticity are often abnormally activated in the TME. The IL-33/ST2 pathway is one important regulatory axis. IL-33 not only promotes mast cell accumulation and pro-inflammatory phenotype but can also downregulate FcϵRI expression at the mast cell precursor stage, affecting their developmental trajectory (41, 50). In cutaneous squamous cell carcinoma, the IL-4/STAT6 pathway interactions between tumor cells and mast cells trigger excessive activation of extracellular signal-regulated kinase/protein kinase B (ERK/Akt), inducing mast cell degranulation and IL-17A secretion, promoting tumor progression (51). Transforming growth factor-beta TGF-β signaling is also critical. In intestinal fibrosis models, mast cell tryptase synergizes with TGF-β1 to promote fibroblast-to-myofibroblast differentiation via Akt and Smad2/3 signaling pathways, exacerbating tissue remodeling (57). Additionally, innate immune receptor signals and the hypoxic microenvironment regulate mast cell cytokine synthesis and mediator secretion through specific molecular elements and signaling pathways, making them core sensors for perceiving microenvironmental changes and controlling tissue homeostasis (58). The crosstalk among these pathways constitutes a fine-tuned regulatory network that determines the final phenotype and functional output of mast cells in a specific microenvironment.

Current State: Multiple signaling pathways (IL-33/ST2, IL-4/STAT6, TGF-β) regulate mast cell phenotype in preclinical models.

Knowledge Gaps: The relative importance of these pathways in human TNBC, and their potential interactions, are unknown.

Future Directions: Human TNBC tissue analysis and organoid co-culture studies could determine which pathways dominate in the human TME and whether they represent therapeutic targets.

### 3.3 apMC as a predictor and intervention target for immunotherapy response

A core challenge in TNBC immunotherapy is identifying new mechanisms of anti-programmed cell death protein-1 (PD-1) resistance and effectively assessing their efficacy and safety in humans. The identification of apMCs opens new avenues for predictive biomarkers and therapeutic interventions. Wu et al. demonstrated that high levels of intra-tumoral apMCs were correlated with enhanced clinical benefit from anti-PD-1 therapy in TNBC patients (14). Mechanistically, apMCs are primarily located within tertiary lymphoid structures, capable of efficient direct and cross-presentation of antigens and expressing co-stimulatory molecules, thus playing a key role in tumor immune control. This clinical finding suggests that the proportion and functional state of apMCs could serve as a novel biomarker for predicting immunotherapy response, potentially more informative than PD-L1 expression alone.

**Figure 2:**
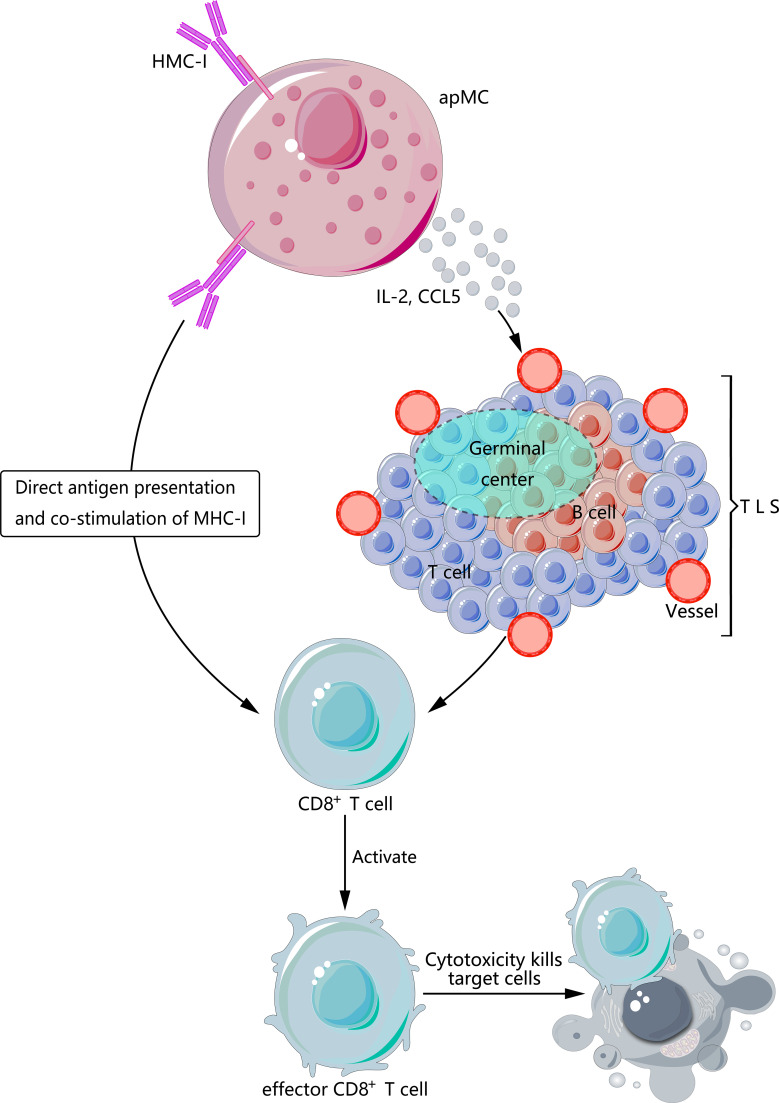
This schematic diagram summarizes a new immunotherapy strategy based on antigen-presenting mast cells (apMCs). Drugs (such as cromolyn sodium) or cytokines are used to ‘re-educate’ mast cells in the tumor microenvironment, transforming them from a pro-tumor phenotype into apMCs with antigen-presenting functions. Activated apMCs directly present tumor antigens to CD8^+^ T cells via MHC-I molecules and secrete key cytokines (such as IL-2) and chemokines (such as CCL5). These signals work together to strongly activate tumor-specific CD8^+^ T cells, driving their proliferation and differentiation into effector cells, ultimately achieving efficient recognition and killing of tumor cells.

The success of the phase 2 trial with cromolyn sodium has spurred interest in other strategies to modulate mast cell plasticity. For example, preclinical studies have shown that IL-33 can promote MHC-II expression on mast cells in mouse models (50), suggesting a potential avenue for further investigation. However, these findings remain preliminary and require validation in human systems. More speculative approaches, such as designing vaccines or adoptive cell therapies based on apMCs, are currently at a conceptual stage and would necessitate extensive preclinical development before clinical translation. These strategies aim to directly enhance apMC function, thereby activating and maintaining effective anti-tumor T cell responses (**Figure 2**).

However, translating apMCs into clinically feasible intervention targets still faces significant challenges. The primary challenge is how to precisely regulate their plasticity, ensuring interventions can stably induce or maintain the apMC phenotype while avoiding their shift towards harmful phenotypes with pro-tumor or pro-fibrotic effects. Second, any intervention aimed at enhancing immune cell activity may cause off-target effects, such as uncontrolled systemic immune activation or allergic reactions, necessitating fine dose control and administration regimens. The sample size was small, the trial was single-arm, and the duration of response was not reported. These limitations highlight the need for larger, randomized trials to confirm efficacy and to identify biomarkers that predict which patients are most likely to benefit. Nevertheless, a phase 2 clinical trial (cromolyn combined with anti-PD-1 regimen) in women with anti-PD-1 refractory metastatic TNBC achieved positive results, meeting the pre-specified primary endpoint with a confirmed ORR of 50.0% (14). This clinical translation success case not only validates the feasibility of targeting apMC strategy but also highlights a reverse translational framework from mechanism discovery to clinical validation, providing new precision immuno-oncology insights for overcoming immunotherapy resistance. Based on this initial clinical signal, future research should focus on elucidating the precise signaling networks regulating apMC polarization and exploring strategies combining it with other immune-modulating approaches to maximize efficacy and minimize risk.

Current State: A clinical proof-of-concept exists that targeting mast cell plasticity can overcome ICI resistance in a subset of patients.

Knowledge Gaps: The trial was small and single-arm; predictors of response were not identified; the duration of response is unknown.

Future Directions: Larger, randomized trials are needed, with comprehensive biomarker analysis to identify which patients benefit. Mechanistic studies should elucidate how cromolyn induces MHC-II upregulation.

## 4 Regulation of mast cell function by the metabolic microenvironment—mechanisms and interventional opportunities

### 4.1 Overview of TNBC metabolic reprogramming

TNBC is characterized by profound metabolic reprogramming to meet the bioenergetic and biosynthetic demands of its rapid proliferation. One core feature of this reprogramming is a strong “Warburg effect,” where TNBC cells preferentially undergo aerobic glycolysis to rapidly generate energy and metabolic intermediates even under oxygen-sufficient conditions (15). Studies show that key glycolytic enzymes such as lactate dehydrogenase A (LDHA), pyruvate kinase M2 (PKM2), and glucose transporters (e.g., GLUT1, GLUT3) are significantly upregulated in TNBC cells, driving enhanced glycolytic flux (59, 60). Beyond glycolytic dependency, TNBC also exhibits significant “glutamine addiction.” Glutamine, as an important nitrogen and carbon source, is catalyzed by glutaminase (GLS) to generate glutamate, entering the tricarboxylic acid (TCA) cycle or used for synthesizing the antioxidant glutathione (GSH), crucial for maintaining cellular redox balance and biosynthesis (9, 61). Research confirms that targeting glutamine metabolism (e.g., using the glutaminase inhibitor CB839) can reshape the immunosuppressive TME and enhance anti-tumor immune responses (62). Simultaneously, lipid metabolism in TNBC cells undergoes significant alterations, manifested as enhanced fatty acid synthesis. The oncogene MYC and its derived circular RNA circMyc drive fatty acid synthesis by regulating the transcription and mRNA stability of sterol regulatory element-binding protein 1 (SREBP1), upregulating downstream lipogenic enzymes, thereby promoting TNBC progression (63). Fatty acid binding protein 4 (FABP4)-mediated fatty acid uptake plays a key role in regulating TNBC lipid metabolism and cancer stem cell activity (64). In brain-metastatic TNBC, downregulation of retinoic acid receptor responder 2 (RARRES2) reprograms lipid metabolism via the PTEN-mTOR-SREBP1 signaling pathway, increasing glycerophospholipid levels, promoting cancer cell survival in the unique brain microenvironment (65).

Mitochondrial function remodeling is another core aspect of TNBC metabolic reprogramming. TNBC cells exhibit metabolic heterogeneity, with mitochondrial morphology and function differing among subtypes or stages. Metastatic TNBC is associated with fused mitochondrial morphology and enhanced glycolysis and lipogenesis metabolism (66). As a key regulator of inter-organelle signaling, the lysosomal calcium release channel TRPML1 impairs mitochondrial respiration upon knockdown, reprograms cellular metabolism, and triggers mitochondrial fragmentation, thereby enhancing the sensitivity of TNBC cells to chemotherapy drugs (67). Additionally, mitochondrial-associated proteins like ATP5MF are highly expressed in non-BRCA1/2 mutated TNBC; its silencing impairs mitochondrial function and inhibits tumor growth, suggesting its important role in energy metabolism (68). This metabolic reprogramming is driven not only by intrinsic oncogenes (e.g., MYC, EGFR) (69) but also under strong selection pressure from the external hypoxic and nutrient-deprived TME (70).

The vast amounts of intermediates and end-products (e.g., lactate, ketone bodies, reactive oxygen species) produced by metabolic activities directly spill into the microenvironment. Lactate accumulation causes acidification, while competitive consumption of glucose, glutamine, and tryptophan, coupled with the production of immunosuppressive metabolites like kynurenine and adenosine, creates a chemical terrain that is hostile to effector immune cells (71, 72). These metabolic adaptations become important mediators of intercellular communication, profoundly influencing immune cell function and shaping an immunosuppressive TME, ultimately leading to immunotherapy resistance (9, 73).

Current State: The metabolic landscape of TNBC is well characterized at the molecular level.

Knowledge Gaps: The spatial heterogeneity of metabolism within tumors, and how it evolves during treatment, is poorly understood.

Future Directions: Metabolomics imaging and spatial transcriptomics could map metabolic heterogeneity and identify metabolic niches that support immunosuppressive cells.

### 4.2 Systemic regulation of immune cell function by metabolites

Metabolites in the tumor microenvironment systemically regulate immune cell function through various mechanisms, profoundly affecting the outcome of anti-tumor immune responses. High concentrations of lactate, a typical product of tumor glycolytic metabolism, not only cause microenvironmental acidification but also directly inhibit the function of effector immune cells. Studies show that lactate, by lowering microenvironmental pH, can inhibit the cytotoxic function of T cells and natural killer (NK) cells. Additionally, lactate can inhibit T cell nuclear factor NFAT signaling transduction and upregulate programmed cell death protein-1 (PD-1) expression, thereby exacerbating T cell exhaustion. Simultaneously, lactate promotes the expansion and function of Tregs and drives TAMs to polarize towards the immunosuppressive M2-like phenotype, collectively shaping an immunosuppressive microenvironment (16). This lactate-mediated immunosuppression is a key mechanism of tumor immune escape.

Abnormal lipid metabolism is another prominent feature of the TME, and specific lipid molecules produced have strong immunomodulatory effects. Lipid mediators released by tumor cells, such as prostaglandin E2 (PGE2), are known immunosuppressants (16). Cholesterol and its metabolites play complex roles in immune regulation. Cholesterol metabolism dysregulation, particularly its oxidized metabolites oxysterols, can affect immune cell localization, migration, and function (74). In the TME, cancer cells reprogram cholesterol metabolism not only to meet their own proliferation needs but also release cholesterol metabolites to inhibit effector T cell function and promote the activity of immunosuppressive cells like Tregs and M2 macrophages, thereby promoting immune escape (75).

Amino acid competition is a core aspect of the metabolic interplay between tumors and immune cells. The excessive consumption of essential amino acids like glutamine, tryptophan, and arginine by tumor cells deprives effector immune cells like T cells of necessary nutrients, leading to their functional exhaustion and impaired proliferation (16). Tryptophan metabolism is particularly critical. Indoleamine 2,3-dioxygenase (IDO), highly expressed by tumor cells and certain immune cells, breaks down tryptophan into kynurenine. The latter not only depletes tryptophan in the microenvironment but its accumulation itself can directly inhibit T cell function and promote Treg differentiation via pathways like aryl hydrocarbon receptor (AhR) activation (76). Arginine depletion similarly impairs T cell receptor signaling and proliferative capacity. This nutrient deprivation forces T cells into a dysfunctional state, while immunosuppressive cells (e.g., Tregs, M2 macrophages) often adapt better to this nutrient-scarce microenvironment, gaining a competitive advantage (77). Microbial metabolites of tryptophan (e.g., indole derivatives) can also regulate T cell and NK cell activity via the AhR pathway, affecting immune checkpoint blockade therapy efficacy (78). In summary, tumor-derived metabolites systematically reshape the immune microenvironment through multiple pathways like acidification, lipid signaling, and nutrient deprivation, suppressing anti-tumor immunity and forming an important basis for immunotherapy resistance.

### 4.3 Regulation of mast cell phenotype and function by metabolites

The metabolic landscape of the TIME specifically shapes the phenotype and function of infiltrating MCs (**Table 2**), creating a direct link between the two pillars of resistance.

**Table 2:** Key metabolites and their roles in the regulation of mast cell function.

Key metabolites	Source	Direct regulatory effects on mast cells	Reference
Lactate	High concentration accumulation in hypoxic tumor regions	Inhibits differentiation of mast cells towards anti-tumorigenic phenotypes (e.g., apMC), downregulates MHC-II and co-stimulatory molecules	(79, 80)
Saturated Fatty Acids (e.g., Palmitic Acid)	Lipid metabolism products	Inducing mast cells to produce pro-tumorigenic cytokines	(80)
Short-Chain Fatty Acids(e.g., Butyric Acid)	Metabolites produced by the intestinal microbiota	Inhibiting HDACs, regulating immune cell function; anti-inflammatory in allergic diseases, complex role in tumor context	(81)
Hypoxia signaling (HIF-1)	Hypoxia feature of TME, Stable HIF-1α	Systemically regulates the expression of multiple mediators and enhances the immunosuppressive function of mast cells	(79)

lactate, the main end-product of glycolysis, accumulates in large quantities in hypoxic tumor regions and has been shown to significantly affect MC function. Studies indicate that lactate can promote the release of key mediators like vascular endothelial growth factor (VEGF) and interleukin-6 (IL-6) from MCs (79). This metabolite-driven mediator release pattern enhances the pro-angiogenic and pro-inflammatory (potentially pro-tumor) phenotype of MCs, thereby supporting tumor growth and the formation of an immunosuppressive microenvironment. Simultaneously, evidence suggests that high lactate concentrations may inhibit the differentiation of MCs towards anti-tumor potential phenotypes like apMC, suppressing the expression of MHC-II and co-stimulatory molecules (80).

Specific lipid species also regulate MCs. Saturated fatty acids like palmitic acid may induce MCs to produce a series of pro-tumor cytokines by activating Toll-like receptor 4 (TLR4) signaling (80). This innate immune receptor activation directly triggered by metabolites converts nutritional signals into inflammatory signals, making MCs a key node linking metabolic reprogramming and tumor-associated inflammation. Furthermore, gut microbiota-derived short-chain fatty acids (SCFAs) like butyrate are also shown to regulate the function of various immune cells, including MCs, by inhibiting histone deacetylases (HDACs). Although they mainly exhibit anti-inflammatory properties in allergic diseases, their role in the tumor context may be more complex (81).

Hypoxia is a core feature of the TME. The stabilization of its key regulator, hypoxia-inducible factor-1alpha HIF-1α, in MCs can systematically regulate the expression of various mediators (79). Under hypoxic conditions, HIF-1α directs MC function towards adaptation to the hypoxic microenvironment, often associated with enhanced immunosuppressive functions, such as promoting the production of immunomodulatory mediators. In summary, metabolites like lactate, fatty acids, and hypoxic signals in the TME specifically shape MCs into phenotypes supporting tumor progression through different molecular mechanisms. Targeting these metabolite-MC axes may provide new intervention ideas for overcoming immunotherapy resistance in TNBC. However, these findings are primarily derived from *in vitro* and animal studies. Direct evidence in human TNBC is lacking, and whether modulating these pathways would yield therapeutic benefit in patients remains to be determined.

Current State: Preclinical evidence indicates that lactate, fatty acids, and hypoxia can modulate mast cell function, but direct evidence in human TNBC is limited.

Knowledge Gaps: The relevance of these findings to human mast cells in the TME is unknown. Whether metabolite concentrations in human TNBC reach levels that affect mast cell function has not been established.

Future Directions: Human mast cells should be exposed to metabolite concentrations measured in human TNBC interstitial fluid to determine physiological relevance. Organoid co-culture systems with controlled metabolite levels could test causality.

### 4.4 Feedback regulation of the metabolic microenvironment by mast cells

Activated mast cells profoundly influence the metabolic state of tumors and surrounding cells by releasing a series of bioactive mediators, thereby shaping a dynamic microenvironment supportive of tumor progression and immune evasion. In breast cancer, particularly TNBC, this feedback regulation mechanism is especially critical. Research shows that tumor-infiltrating mast cells (TIMCs) play a central regulatory role in the TIME, capable of exerting unique anti-tumor or pro-tumor effects by enriching and activating other immune cell populations (82). This regulatory effect is partly achieved by altering the local metabolic environment. In hormone receptor-positive (HR+) breast cancer, progesterone receptor (PGR) can transcriptionally upregulate KIT ligand (KITLG), thereby recruiting and activating mast cells and promoting the production of mast cell-derived granulin (GRN). GRN attenuates TNFα-induced breast cancer cell apoptosis by competitively binding TNFR1. This process directly interferes with the mitochondrial apoptosis pathway of tumor cells, exemplifying the direct impact of mast cell mediators on tumor cell metabolic fate (45). This PGR-KITLG signal-driven tumor-mast cell regulatory feedback loop is a typical example of tumor cells utilizing mast cells to establish an anti-apoptotic microenvironment.

Furthermore, cytokines and chemokines released by mast cells can broadly regulate the immune-metabolic landscape in the TME. In TNBC, High potassium channel tetramerization domain containing 5 (KCTD5) expression predicts poor prognosis and may shape an immunosuppressive microenvironment by affecting immune cell infiltration, including mast cells (83). This microenvironment is characterized by metabolic reprogramming, potentially suppressing effector T cell function while supporting the survival and activity of immunosuppressive cells like M2 macrophages. Research confirms that in the analysis of serum-derived extracellular vesicles (EVs) from breast cancer patients, a high EV score correlates with enrichment of anti-inflammatory M2 macrophages and mast cells, while these samples have low stromal tumor-infiltrating lymphocyte (TIL) counts, suggesting mast cells may participate in establishing a metabolic and immunosuppressive state that inhibits adaptive immune responses (84).

More importantly, the metabolic feedback regulation by mast cells is closely related to therapy resistance. After neoadjuvant chemotherapy (NAC), the infiltration of specific immune cell populations, including mast cells (e.g., Treg, neutrophils, macrophages), increases. This change varies by breast cancer subtype and may collectively contribute to therapy resistance (85). In TNBC, targeting the PI3K-mTOR pathway can overcome immunotherapy resistance. One mechanism involves profoundly altering the TIME, reducing adaptive immune phenotypes associated with immunotherapy resistance (like exhausted T cells and Tregs) and pro-tumor innate immune populations, which include mast cells (86). This indicates that intervening in the metabolic pathways driving mast cell activation and function (like PI3K-mTOR) can disrupt the immunosuppressive metabolic microenvironment they maintain, thereby restoring anti-tumor immunity. In summary, through the release of mediators and cytokines, mast cells form a complex metabolic feedback network with tumor cells and other immune cells, jointly maintaining a homeostasis favorable for tumor growth and immune evasion. Targeting key nodes in this feedback loop provides new strategic directions for reversing TNBC immunotherapy resistance.

Current State: Evidence supports bidirectional crosstalk: mast cells both respond to and shape the metabolic microenvironment.

Knowledge Gaps: The specific mediators and mechanisms by which mast cells alter metabolism in human TNBC are poorly defined.

Future Directions: Co-culture systems with metabolic flux analysis could identify how mast cells alter tumor cell metabolism. *In vivo* studies with mast cell-deficient models could establish causality.

## 5 Organoid platforms-a revolutionary platform for deciphering complex interactions

### 5.1 Unique advantages and current limitations of organoid models in TNBC research

Organoid models have emerged as revolutionary three-dimensional (3D) cell culture systems that offer unique advantages for deciphering the complex mechanisms of immunotherapy resistance in TNBC. First and foremost, patient-derived TNBC organoids (PDOs) can faithfully preserve the genetic heterogeneity, histological architecture (such as glandular structures), and key driver mutations of the parental tumor *in vitro*, making them a highly clinically relevant model compared to traditional two-dimensional cell lines that often undergo phenotypic drift. This high degree of fidelity underpins their value in predicting patient-specific treatment responses and survival outcomes (87). Secondly, organoid technology allows for long-term expansion and cryopreservation, facilitating the establishment of large-scale, reusable biobanks. This provides a convenient and scalable platform for high-throughput drug screening, genetic manipulation, and functional genomics studies (88). The integration of multi-omics analyses with PDOs has been effective in identifying candidate therapeutic targets and elucidating drug mechanisms of action (89). Thirdly, compared to 2D culture, 3D organoid models more accurately simulate the *in vivo* cell-cell interactions and critical microenvironmental features such as metabolic and oxygen gradients, including the formation of hypoxic cores (88). Maintaining this 3D spatial architecture is crucial for studying tumor growth, multicellular spatial relationships, and responses to therapies. Furthermore, organoids support advanced, high-throughput imaging and analysis pipelines that can deconvolute complex organoid dynamics at cellular resolution, enabling sophisticated 3D image-based pharmacological testing (90).

When compared with conventional animal models, PDOs present a distinct balance of advantages and limitations that must be carefully weighed in translational TNBC research. From a feasibility perspective, establishing PDOs from patient tumor biopsies or surgical specimens is typically faster and more cost-effective than generating genetically engineered mouse models (GEMMs) or patient-derived xenografts (PDXs). Successful establishment of TNBC organoids generally requires only small tissue fragments or core-needle biopsies, yielding sufficient epithelial cells for 3D culture initiation within weeks (18). In contrast, PDX models necessitate larger tumor volumes, immunocompromised host animals, and 3–6 months of engraftment and passaging before experimental use, substantially increasing time and monetary costs. GEMMs, while powerful for studying tumor initiation and progression in an immunocompetent setting, require extensive genetic manipulation, breeding programs, and months to years of development, limiting their scalability for high-throughput screening (91). Regarding comparability and human relevance, PDOs offer superior fidelity to the parental tumor’s genetic landscape, histopathology, and drug response patterns, as they are derived directly from human tissue without interspecies adaptation or murine stromal replacement (18). Animal models, conversely, are constrained by fundamental interspecies differences in immune architecture, metabolism, and drug pharmacokinetics, which can lead to discordant responses between preclinical efficacy and clinical outcomes. Nevertheless, animal models retain unique strengths that organoids cannot fully replicate: they preserve systemic physiological contexts (including endocrine, neural, and circulatory inputs), enable longitudinal monitoring of metastatic dissemination, and facilitate the study of organism-level drug toxicities (92). Thus, rather than viewing organoids and animal models as mutually exclusive, the field is increasingly recognizing their complementary roles—with PDOs serving as rapid, patient-specific platforms for mechanistic dissection and drug prioritization, and animal models providing indispensable *in vivo* validation for the most promising candidates identified in organoid screens (93).

However, organoid models also possess significant limitations for TNBC research. A primary constraint is that traditional organoid culture systems often lack critical components of the native tumor microenvironment, such as functional immune cells, vascular networks, and cancer-associated fibroblasts (CAFs) (88). Although some advanced co-culture systems have been developed, fully recapitulating the intricate immune and vascular networks *in vivo* remains a significant challenge (94). Secondly, the extracellular matrix (ECM) components (e.g., Matrigel^®^) essential for organoid growth are difficult to standardize, and batch-to-batch variations can affect experimental reproducibility. The regulation of ECM composition and stiffness, which influences organoid invasiveness and drug resistance, highlights both its importance and the associated standardization challenges (95). Thirdly, as *in vitro* models, organoids inherently lack systemic influences present *in vivo*, such as hormonal regulation, neural input, and systemic immune responses. While powerful for studying certain processes like radiation-induced cell recruitment, mammary organoids cannot fully simulate the complex systemic inflammation and immune cell recruitment that occurs post-radiation *in vivo* (96). Finally, although PDOs show great promise in predicting responses to combination therapies like chemotherapy plus ICIs, the technical and temporal challenges associated with establishing robust co-culture systems with autologous immune cells currently limit their immediate application in real-time clinical decision-making (97).

Current State: Organoid technology is mature and validated for preserving tumor characteristics and drug testing.

Knowledge Gaps: Standardization across laboratories remains a challenge. The optimal conditions for maintaining specific immune cell types in co-culture are not fully defined.

Future Directions: Development of standardized protocols and quality control metrics would enable broader use and cross-study comparability.

### 5.2 Constructing organoid systems supporting mast cell survival and function

Building immunocompetent TNBC organoid systems that support the study of mast cells (MCs) is a key prerequisite for deeply deciphering their function and plasticity within the TME. The foundation of this system lies in establishing patient-derived organoids that faithfully reflect the heterogeneity and biological characteristics of patient tumors. Research shows that PDOs established from fresh TNBC tissue or patient-derived xenograft models can maintain tumor cell stemness, key driver mutations, and histopathological features in 3D culture, providing a highly physiologically relevant platform (97). To ensure long-term culture and phenotypic stability of organoids, optimized culture media are typically required, containing specific growth factors and small molecule inhibitors to simulate*in vivo* niche signals, maintain organoid proliferative capacity, and prevent unintended differentiation or phenotypic drift.

The core step in building an immunocompetent system is the successful embedding of functional mast cells. Mast cells, as tissue-resident immune cells, originate from bone marrow precursors and undergo final differentiation in peripheral tissues under local signals (54). Therefore, mast cell precursors isolated from patient autologous peripheral blood or healthy donors, or directly isolated tumor-infiltrating mast cells, can be introduced into the organoid culture. To effectively induce and maintain mast cell survival, phenotype, and function, appropriate cytokine combinations must be provided. Stem cell factor (SCF) is a key factor for mast cell survival and proliferation, while IL-33 can significantly influence their activation and functional state (50, 98). Adding these cytokines simulates the “education” effect of the TME on mast cells, promoting their interaction with tumor organoids.

The successful application of this co-culture system depends on optimizing several key parameters. First, the optimal ratio and timing for co-culturing organoids and mast cells must be determined to ensure the viability and effective interaction of both cell types. Second, media composition requires careful optimization, for example, avoiding serum that may contain high histamine concentrations to prevent background interference in measuring mast cell mediator release. Finally, a set of real-time, quantitative monitoring indicators must be established to assess mast cell status in the co-culture system. This includes periodic detection of classic mast cell surface markers (CD117, FcϵRI) via flow cytometry to confirm identity and activation state, alongside molecules like MHC-II to assess antigen-presenting potential (99). At the functional level, assays to quantify mediator release from mast cells (e.g., tryptase, histamine, TNF-α) under co-culture conditions are essential. Furthermore, this system can be combined with T cells for tripartite co-culture. By detecting T cell proliferation, activation markers (CD69, CD25), and effector cytokine *e.g.*, *IFN-γ* secretion, the regulatory function of mast cells in shaping anti-tumor immune responses can be assessed, comprehensively deciphering their complex role in the TNBC immune microenvironment.

Advanced bioengineering techniques further enhance these models. Microfluidic “organ-on-a-chip” systems allow the creation of chemokine gradients to simulate immune cell migration and infiltration (100). Even more precise is the use of acoustic manipulation techniques, such as “acoustic virtual 3D scaffolds,” which enable label-free, high-precision assembly of immune cells (like T cells and MCs) in direct contact with organoids in 3D space. This technology has been shown to dramatically enhance the efficiency of immune synapse formation and T cell activation compared to traditional methods (101).

### 5.3 Application of organoids in studying apMC-metabolism interactions

Organoid models provide a unique and controllable platform for in-depth investigation of the interaction between antigen-presenting mast cells (apMCs) and tumor metabolic reprogramming in TNBC. For mechanistic exploration, genetically editing organoids or using metabolic modulators allows precise assessment of their impact on immune cell phenotype and function in co-culture systems. Research confirms that changes in metabolites can directly affect immune responses. One study found that accumulation of the metabolite GDP-mannose (GDP-M) suppresses homologous recombination repair in TNBC, and this process was validated in patient-derived organoid models, suggesting metabolic modulation can reshape the TME (102). Another strategy involves using compounds like isocucurbitacin B (IB) to target STAT3, disrupt the TCA cycle, induce ferroptosis, and alter tumor cell immunogenicity (103). These studies provide a methodological foundation for simulating lactate metabolic changes in organoid-mast cell co-culture systems by knocking out LDHA or adding metformin and then observing the impact on mast cell polarization towards the apMC phenotype and their antigen-presenting capacity.

In therapeutic simulation, organoid models are ideal tools for assessing the synergistic effects of combination strategies. Research shows that combining metabolic interventions with ICIs can significantly enhance anti-tumor immunity. In TNBC organoid-T cell co-culture systems, supplementing GDP-M enhanced the efficacy of anti-PD-1 antibodies when combined with a PARP inhibitor (102). Similarly, the γ-secretase modulator sulindac sulfide (SS) inhibited TNBC organoid growth without affecting T cell Notch signaling and showed significant synergistic anti-tumor activity with anti-PD-1 immunotherapy (104). These findings support using organoid-immune cell co-culture systems to assess the synergistic effects of simultaneously applying anti-PD-1 antibodies and specific metabolic interventions or phenotype modulators on organoid growth inhibition and T cell activation. This simulation can directly reflect the interaction between metabolic reprogramming and immune checkpoint blockade, providing preclinical evidence for designing combination regimens to overcome immunotherapy resistance.

In the field of biomarker discovery, performing multi-omics analysis on organoid-immune cell co-culture systems is a powerful means to mine signature profiles associated with treatment response. Several studies have successfully identified key predictive molecules by combining organoid models with multi-omics technologies. Integrating spatial transcriptomics and chromatin accessibility analysis revealed that high expression of tumor-specific MHC-II (tsMHC-II) correlates with an immune-rich microenvironment and better immunotherapy response in TNBC (105). In patient-derived organoid-T cell co-culture systems, interferon-induced CD8+ T cell senescence was linked to immunotherapy non-response, and metabolomics analysis revealed features like excessive NAD+ consumption (106). Furthermore, engineered exosomes siT/MOF@EVs, when targeting TNBC organoids, can simultaneously overcome multi-level heterogeneities like stemness, upregulated immune checkpoints, and metabolic plasticity through membrane-cytoplasm-mitochondria cascade targeting, with efficacy validated in patient-derived organoid xenograft models (107). These paradigms demonstrate that through transcriptomic, metabolomic, and proteomic analysis of organoid-mast cell co-culture systems under different interventions, it is entirely possible to systematically screen multi-omics biomarkers closely related to apMC abundance, metabolic state, and sensitivity to combination therapies, thereby providing new decision-making bases for precision immunotherapy in TNBC.

## 6 Mechanism-based multidimensional therapeutic strategies

### 6.1 Therapeutic strategies targeting apMC

Developing strategies to target and reprogram mast cells, particularly towards anti-tumor phenotypes (apMC), is a promising approach to overcome immunotherapy resistance. One key strategy involves the direct pharmacological reprogramming of mast cell plasticity. The classic anti-allergy drug sodium cromolyn (cromolyn sodium) has emerged as a lead candidate. Beyond its known role as a mast cell stabilizer, recent research reveals its capacity to induce an apMC phenotype characterized by upregulated MHC-II and co-stimulatory molecule (CD80/CD86) expression (14). In apMC-organoid-T cell tripartite co-culture models, cromolyn-induced apMCs form stable immunological synapses with T cells, leading to potent T cell activation, clonal expansion, and enhanced tumor cell killing. This provides a strong rationale for combining cromolyn with PD-1/PD-L1 inhibitors, creating a logical therapeutic loop of “optimized antigen priming (by apMC) + released inhibition (by ICI)”. A phase 2 clinical trial in anti-PD-1 refractory metastatic TNBC combining cromolyn with anti-PD-1 therapy reported a promising ORR of 50.0% (14), offering clinical proof-of-concept.

Other strategies focus on promoting apMC polarization and preventing polarized apMCs from undergoing phenotypic reversion in the suppressive TME via cytokine modulation. Cytokines such as IL-4 and IL-33 have been shown to promote MHC-II expression on mast cells, directing them towards an apMC phenotype (50). Therefore, the use of IL-33 agonists or strategies to enhance local IL-4 signaling represents another intervention avenue. Conversely, inhibiting signals that suppress apMC function is equally important. The Notch signaling pathway plays an important role in TNBC progression and immune regulation including confer the functions as an antigen-presenting cell on mast cells (108). Research found that the non-steroidal anti-inflammatory drug sulindac sulfide (SS), as a γ-secretase modulator, can specifically inhibit Notch1 cleavage in TNBC cells without affecting Notch signaling in T cells, and showed significant synergistic anti-tumor activity with anti-PD-1 immunotherapy in TNBC organoid and *in vivo* models (104). This provides an example for developing targeted phenotype stabilizers that avoid broad immunosuppression. Such drugs may function by blocking specific intracellular signals that push apMCs towards inhibitory phenotypes.

**Figure 3:**
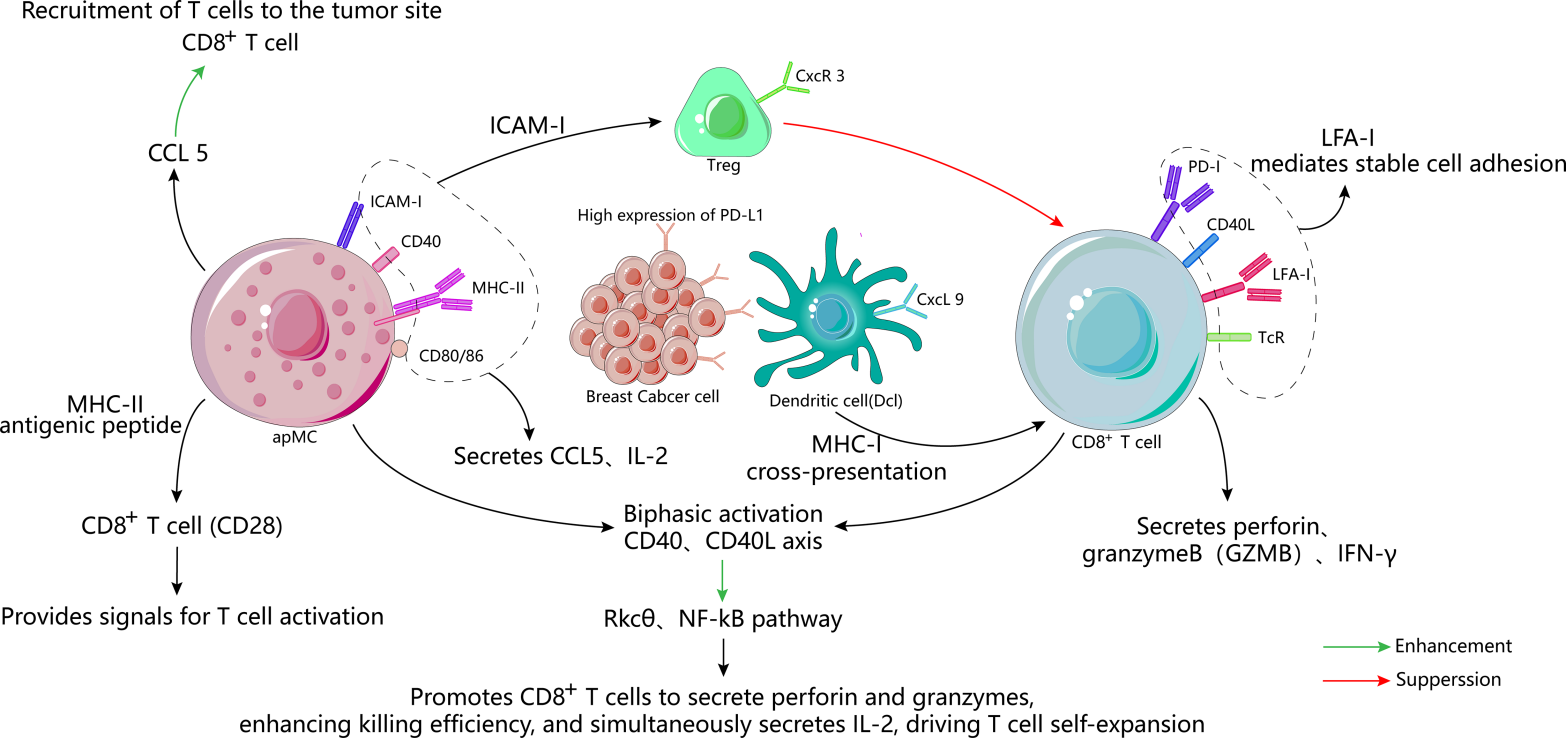
This schematic diagram details the formation of functional immunological synapses between reprogrammed apMCs and CD8^+^ T cells, as well as their activation pathways. The main steps include: (1) cell adhesion: establishing stable contact via ICAM-1/LFA-1; (2) antigen recognition and co-stimulation: T cell receptors (TCRs) recognize antigen peptides presented by MHC-II on apMCs, while CD28 binds to CD80/CD86 on apMCs, providing key co-stimulatory signals; (3) synergistic signal activation: CD40 on apMCs interacts with CD40L on T cells, further amplifying the activation signal; (4) intracellular signaling: these signals converge to recruit and activate PKCθ within T cells, thereby strongly driving signaling pathways such as NF-κB; (5) functional output: leading to explosive secretion of effector molecules (perforin, granzyme B, IFN-γ) and growth factors (IL-2) by T cells, resulting in clonal expansion and potent cytotoxicity. The diagram also illustrates the cross-presentation function of dendritic cells (DCs), as well as the inhibitory context formed by regulatory T cells (Tregs) and tumor cells with high PD-L1 expression, highlighting the necessity for combined intervention.

For stubborn, non-reprogrammable pro-tumor mast cell subsets, strategies for selective clearance agents are being explored. This includes developing antibody-drug conjugates (ADCs) or bispecific antibodies targeting surface markers highly expressed on these subsets (e.g., specific protease profiles). CAR-NK cell therapy targeting the tumor-associated antigen mesothelin (MSLN) has shown high killing efficacy against TNBC cells in preclinical models, including patient-derived primary cells and organoid models (109). Specific Toll-like receptor (TLR) ligands have been shown to be effective immune modulators. One study developed an *in situ* dendritic cell (DC) vaccine (HELA-Exos) delivering the TLR3 agonist Hiltonol via engineered exosomes. In TNBC mouse models and patient-derived organoids, this formulation effectively induced immunogenic cell death of tumor cells (110). Although these studies did not focus on mast cells, the principle similarly applies to targeted therapy against specific mast cell subsets: designing specific antibody carriers recognizing surface markers highly expressed (like CD200R) to precisely deliver cytotoxic drugs or effector immune cells to target cells. Another study designed a novel extracellular vesicle (EV) delivery system siT/MOF@EVs that can act as a “decoy” binding to multiple immune checkpoint ligands on tumor cell (including cancer stem cell) surfaces, thereby blocking inhibitory signals (107). This multi-target blockade strategy, if combined with specific antibodies against pro-tumor mast cells, holds promise for efficient clearance of this stubborn subset, with effects validated in patient-derived organoid xenograft (PDOX) models. In summary, combined with organoid models for high-throughput screening and functional validation, drug development targeting different aspects of mast cell plasticity-polarization induction, phenotype stabilization, and specific clearance-will provide new intervention weapons for overcoming TNBC immunotherapy resistance (**Figure 3**).

Current State: A clinical proof-of-concept exists that targeting mast cell plasticity can overcome ICI resistance in a subset of patients.

Knowledge Gaps: The trial was small and single-arm; predictors of response were not identified; the duration of response is unknown.

Future Directions: Larger, randomized trials are needed, with comprehensive biomarker analysis to identify which patients benefit. Mechanistic studies should elucidate how cromolyn induces MHC-II upregulation.

### 6.2 Metabolic intervention strategies and synergistic design with apMC targeting

Given the suppressive effect of metabolites like lactate on apMC function, metabolic interventions hold great promise for synergizing with apMC-targeted therapies. The goal is not merely to starve tumor cells but to selectively reshape the metabolic TIME to “liberate” immune cells, including apMCs. The synergistic logic is compelling: metabolic modulators can first “normalize” the TIME, alleviating the metabolic inhibition on immune cells and creating favorable conditions for apMC differentiation. Subsequently, apMC-targeted agents (e.g., cromolyn) or ICIs can more effectively activate anti-tumor immunity.

Glycolysis is a core energy metabolism pathway in TNBC cells, and the activity of key enzymes like hexokinase 2 (HK2) and signaling pathways (e.g., PI3Kδ/γ or AKT) is closely related to tumor progression and the immunosuppressive microenvironment. Studies show that inhibiting HK2 can not only directly inhibit TNBC cell proliferation but also reshape the TIME. The HK2 inhibitor 3-BrPA can reduce granulocyte colony-stimulating factor (G-CSF) expression in 4T1 cells, thereby decreasing MDSC development, enhancing T cell immunity, and prolonging survival in tumor-bearing mice. Additionally, the combination of 3-BrPA with anti-PD-L1 therapy showed synergistic effects in the 4T1 tumor-bearing mouse model, providing new ideas for combining glycolytic targeting with immune checkpoint blockade therapy (111). The accumulation of lactate from tumor glycolysis acidifies the TME and directly inhibits immune cell function. Inhibitors of lactate transporters MCT1/4 (e.g., AZD3965) can reduce extracellular lactate accumulation and alleviate TME acidification (112). In preclinical models, combining MCT inhibitors with ICIs enhances anti-tumor immunity. In organoid models, such interventions could be tested for their additional benefit in promoting apMC differentiation and function. Dichloroacetate (DCA) and TPP-CuET loaded SeqGel, which redirects metabolism from glycolysis to oxidative phosphorylation, similarly reduces lactate production and could create a permissive environment for antigen processing and presentation to T cells (113). These findings support the potential of targeting key glycolytic nodes to reverse immunosuppression and enhance therapeutic effects.

Drug development targeting lipid metabolism is another frontier in intervening the TNBC immune microenvironment. Dysregulation of fatty acid metabolism plays a key role in breast cancer, particularly TNBC (114). FABP4-mediated lipid metabolism was shown to promote TNBC progression and breast cancer stem cell activity. In the MMTV-Wnt1 spontaneous TNBC model and TNBC patient-derived xenograft models, knocking down or inhibiting FABP4 activity significantly suppressed tumor progression (64). Modulators of cholesterol metabolism, such as ACAT1 inhibitors, can increase free cholesterol in T cell membranes, potentially enhancing TCR clustering and signaling efficiency at the immunological synapse (115). Additionally, COX-1-dependent 15-hydroxyeicosatetraenoic acid (15-HETE) biosynthesis exists in human mast cells, suggesting the complexity of lipid mediator metabolism (116).

Amino Acid metabolism is another metabolic vulnerability of TNBC cells, whose addiction promotes cancer cell proliferation and invasion. Glutaminase (GLS) inhibitors like CB-839, suppresses glutamine metabolism and proliferation in triple-negative breast cancer cells, and exhibits significant antitumor activity both as a single agent and in combination with paclitaxel in TNBC xenograft models (117). Ginsenoside CK was also found to inhibit glutamine consumption and glutamate production in TNBC cells and the SUM159 xenograft mouse model by downregulating glutaminase 1 (GLS1) expression, thereby inducing apoptosis (118). While PEGylated kynureninase degrades kynurenine in the tumor microenvironment, reversing IDO1/TDO-mediated immunosuppression, enhancing polyfunctional CD8+ T-cell infiltration and proliferation, and inhibiting tumor growth, with pronounced efficacy when combined with checkpoint inhibitors or cancer vaccines (119).

In summary, specifically targeting glycolysis, lipid, and Amino Acid metabolism pathways provides a solid theoretical and experimental basis for developing novel combination strategies to reverse the immunosuppressive TNBC microenvironment and overcome therapy resistance.

Current Evidence: Multiple preclinical studies have demonstrated synergy between metabolic interventions and ICIs. In 4T1 models, HK2 inhibitor 3-BrPA combined with anti-PD-L1 prolonged survival (111). In TNBC organoids, GDP-mannose enhanced anti-PD-1 efficacy with PARP inhibition (102). Sulindac sulfide showed synergy with anti-PD-1 in organoid and *in vivo* models (104). Studies on apMC-targeted therapeutic strategies are summarized in **Table 3**.

**Table 3:** Therapeutic strategies targeting antigen-presenting mast cells (apMCs) in TNBC.

No.	Therapeutic strategy	Study content	Study results	Study type	Ref.
Direct Pharmacological Reprogramming of apMCs					
1	Cromolyn sodium + anti-PD-1	Phase 2 clinical trial (NCT05076682) in anti-PD-1 refractory metastatic TNBC. *In vitro*: Cromolyn induces apMC phenotype (upregulated MHC-II, CD80/CD86) in tripartite co-culture models.	Clinical: Confirmed ORR of 50.0%, meeting the pre-specified primary endpoint. *In vitro*: Cromolyn-induced apMCs form stable immunological synapses, leading to potent T cell activation, clonal expansion, and enhanced tumor cell killing.	Phase 2 clinical trial *In vitro* (organoid co-culture)	(14)
2	IL-33/IL-4 cytokine modulation	IL-33 and IL-4 promote MHC-II expression on mast cells, directing them toward an apMC phenotype.	Preclinical evidence in mouse models demonstrates apMC polarization potential; requires validation in human systems.	*In vivo* (mouse models)	(50)
4	Sulindac sulfide (SS; gamma-secretase modulator)	Specifically inhibits Notch1 cleavage in TNBC cells without affecting Notch signaling in T cells; targets Notch pathway that confers antigen-presenting cell functions on mast cells.	Showed significant synergistic anti-tumor activity with anti-PD-1 immunotherapy in TNBC organoid and *in vivo* models.	*In vitro* (organoid) *In vivo* (mouse models)	(104, 108)
Selective Clearance of Pro-tumor MC Subsets					
5	CAR-NK cell therapy (anti-MSLN)	Mesothelin-targeted CAR-NK cells derived from induced pluripotent stem cells.	High killing efficacy against TNBC cells in preclinical models, including patient-derived primary cells and organoid models.	*In vitro* (primary cells) *In vitro* (organoid)	(109)
6	HELA-Exos (engineered exosome DC vaccine)	*In situ* DC vaccine delivering TLR3 agonist Hiltonol via engineered exosomes.	Effectively induced immunogenic cell death of tumor cells in TNBC mouse models and patient-derived organoids.	*In vivo* (mouse models) *In vitro* (organoid)	(110)
7	EV decoy delivery system	Novel extracellular vesicle system acting as a decoy binding to multiple immune checkpoint ligands on tumor cell (including cancer stem cell) surfaces.	Overcame multi-level TNBC heterogeneities via membrane-cytoplasm-mitochondria cascade targeting; efficacy validated in patient-derived organoid xenograft (PDOX) models.	*In vivo* (PDOX)	(107)
Metabolic Interventions Synergizing with apMC Targeting					
8	HK2 inhibitor (3-BrPA)	Inhibits glycolysis in TNBC cells; reduces G-CSF expression in 4T1 cells.	Decreased MDSC development, enhanced T cell immunity, and prolonged survival in tumor-bearing mice; synergistic effect with anti-PD-L1 therapy in the 4T1 mouse model.	*In vivo* (mouse model)	(111)
9	MCT1/4 inhibitor (AZD3965)	Inhibits monocarboxylate lactate transporters MCT1/4 to reduce extracellular lactate accumulation.	Alleviated TME acidification; in preclinical models, combining MCT inhibitors with ICIs enhanced anti-tumor immunity.	*In vitro* (cell lines)	(112)
10	Dichloroacetate (DCA) and TPP-CuET loaded SeqGel	Redirects metabolism from glycolysis to oxidative phosphorylation.	Reduces lactate production, potentially creating a permissive metabolic environment for antigen processing and presentation to T cells	*In vitro* (cell lines) *In vivo* (mouse models)	(113)
11	FABP4 inhibition	Knockdown or pharmacological inhibition of FABP4-mediated fatty acid uptake.	Significantly suppressed tumor progression in MMTV-Wnt1 spontaneous TNBC model and TNBC patient-derived xenograft models.	*In vivo* (mouse, PDX)	(64)
12	ACAT1 inhibitors	Modulators of cholesterol metabolism that increase free cholesterol in T cell membranes.	Potentially enhanced TCR clustering and signaling efficiency at the immunological synapse.	*In vivo* (mouse models)	(115)
13	GLS inhibitor (CB-839)	Suppresses glutamine metabolism and proliferation in triple-negative breast cancer cells	Exhibits significant antitumor activity both as a single agent and in combination with paclitaxel in TNBC xenograft models	*In vitro* (cell lines) *In vivo* (xenograft)	(117)
14	Ginsenoside CK	Downregulates GLS1 expression, inhibiting glutamine consumption and glutamate production in TNBC cells.	Induced apoptosis in TNBC cells and the SUM159 xenograft mouse model.	*In vitro* (cell lines) *In vivo* (xenograft)	(118)
15	PEGylated kynureninase	Degrades kynurenine in the tumor microenvironment, reversing IDO1/TDO-mediated immunosuppression	Enhancing CD8+ T-cell infiltration and proliferation, and inhibiting tumor growth, with pronounced efficacy when combined with checkpoint inhibitors or cancer vaccines	*In vivo* (xenograft)	(119)

Knowledge Gaps: Most studies are preclinical. Clinical validation is lacking. The optimal combinations for specific patient subsets are unknown.

Future Directions: Rational combination design based on mechanistic understanding, followed by biomarker-driven clinical trials, is needed.

## 7 Clinical translation framework: from biomarkers to personalized therapy

### 7.1 Establishment of a biomarker system

Looking toward future clinical application, Translating these multidimensional strategies requires a systematic, biomarker-driven precision framework. Establishing a precise biomarker system is crucial for predicting the response of TNBC patients to therapies targeting activated phenotype mast cells (apMC) or metabolic interventions (120). This system should integrate tissue-based assays and liquid biopsy techniques to comprehensively capture dynamic changes in the tumor microenvironment. At the tissue level, multiplex immunofluorescence can simultaneously detect specific surface markers of apMC, such as MHC-II and CD117 (c-Kit), enabling spatial analysis of their distribution, abundance, and co-localization with immune checkpoint molecules (like PD-L1) within the tumor, providing morphological basis for predicting immunotherapy resistance. Simultaneously, liquid biopsy, as a non-invasive method, can dynamically monitor the activity state of apMC and systemic immune-metabolic changes by detecting mast cell-specific mediators (like tryptase, histamine) or their metabolites in peripheral blood. For example, metabolomic analysis has revealed specific alterations in amino acid, fatty acid, and purine metabolite profiles in the serum of TNBC patients, which may serve as indirect biomarkers for apMC activation and metabolic reprogramming (121). Integrating tissue and liquid biopsy biomarkers into a comprehensive detection panel can assess the immune-metabolic characteristics of tumors from both static and dynamic dimensions, improving accuracy in predicting patient likelihood of response to apMC-targeting or metabolic interventions.

Beyond static biomarker detection, functional biomarkers play a central role in personalized therapy. Patient-derived organoid (PDO) models, particularly organoid-based drug sensitivity testing, provide a powerful functional platform for screening the most effective combination regimens prior to treatment (122). PDOs can highly simulate the tissue structure, gene expression profiles, and drug response heterogeneity of the parental tumor, making them ideal “living biomarkers” (123). High-throughput drug screening using TNBC PDOs can evaluate the effects of different combination regimens (e.g., ICIs combined with apMC-targeting drugs or metabolic modulators). This PDO-based drug testing not only serves as a predictive biomarker guiding clinical selection of the most likely beneficial treatment combinations but can also be used to explore resistance mechanisms and discover new therapeutic targets. Therefore, combining tissue-based molecular markers, liquid biopsy circulating markers, and organoid functional drug sensitivity testing to build a multi-level, dynamic biomarker system is a key strategy for achieving precision immunotherapy and overcoming therapy resistance in TNBC.

### 7.2 New directions in clinical trial design

Addressing the challenge of immunotherapy resistance in TNBC, future clinical trial designs need to be more innovative and mechanism-oriented. Designing Phase I/II clinical trials to explore the combination of drugs targeting activated phenotype mast cells (apMC) with ICIs is an important strategy to overcome resistance (124). Although ICI-chemotherapy combinations have become a standard treatment option for advanced and early-stage TNBC, primary or secondary resistance remains a major obstacle (6). Preclinical studies indicate that immunosuppressive cells in the tumor microenvironment, may promote immune evasion through metabolic reprogramming. Therefore, targeting these cell subsets, particularly apMC, holds promise for reshaping the TME and enhancing ICI efficacy. Such Phase I/II trials should focus on assessing the safety, tolerability, and preliminary anti-tumor activity of combination regimens, laying the groundwork for subsequent confirmatory studies (125).

Incorporating exploratory endpoints in clinical trials, such as changes in the proportion of apMC in tumor biopsies pre- and post-treatment and alterations in serum metabolic profiles, is crucial for validating mechanisms of action. Traditional efficacy endpoints like pathological complete response (pCR) or overall survival (OS), while important, cannot deeply reveal how treatment affects the tumor’s immune and metabolic microenvironment. Through multi-omics analyses, including spatial transcriptomics and metabolomics, the infiltration state of apMC and their interactions with immune cells (like T cells, NK cells) can be dynamically monitored. Simultaneously, monitoring levels of specific metabolites (like lactate, ketone bodies) in serum can assess whether overall tumor metabolic reprogramming is corrected, thereby linking biological findings with clinical outcomes.

Finally, considering incorporating metabolic intervention drugs into basket trial designs for combination therapy is a forward-thinking approach to address TNBC heterogeneity and resistance. Metabolic reprogramming is a core mechanism of tumor immune evasion. Drugs targeting metabolic pathways like cholesterol homeostasis, glycolysis, or mitochondrial function have shown potential synergy with immunotherapy (126, 127). Basket trial designs allow combining metabolic inhibitors (approved or in clinical stages) with different mechanisms of action with ICIs and apMC-targeting drugs, stratifying patients based on biomarkers (e.g., specific gene expression profiles or metabolic enzyme activity). This multi-arm, multi-drug combination trial based on mechanisms can systematically evaluate the efficacy of different combination strategies and rapidly identify the most effective treatment regimens for specific patient populations, thereby accelerating the development of personalized treatment plans.

### 7.3 Conceptualization of personalized treatment pathways

Future personalized treatment pathways for TNBC will deeply integrate cutting-edge *in vitro* model technologies and multi-omics data to construct a closed-loop system from precise diagnosis to dynamic treatment decision-making. The core lies in utilizing patient-derived organoid (PDO) models as *in vitro* “avatars” for high-throughput drug screening and efficacy prediction, thereby guiding clinical medication. Research shows that successfully establishing PDO models from breast cancer tissue, including heavily pre-treated specimens, is feasible (128). These PDO models retain the histological structure, genetic features, and transcriptional profiles of the parental tumor, providing a highly simulated platform for *in vitro* drug sensitivity testing. A study successfully established 75 breast cancer PDO models, and drug sensitivity testing results showed consistency with the clinical responses of matched patients in most cases, demonstrating the potential of the PDO platform in guiding personalized treatment for advanced breast cancer patients (129). Specifically, future diagnostic and therapeutic pathways may start with patient biopsy, followed by establishing PDO co-culture systems containing immune cells (like T cells, macrophages) to simulate the TIME (130). On this platform, multiple combination therapies targeting activated mast cells (apMC) and metabolic reprogramming can be systematically tested to screen the most effective drug combinations for specific patients, achieving “pre-treatment drug testing” *in vitro*, avoiding toxic side effects and time delays from ineffective treatments.

To achieve higher precision in treatment decisions, this pathway needs to integrate patient genomic, transcriptomic, proteomic, and metabolomic data to construct a patient’s “digital twin” model. Multi-omics analysis plays a crucial role in exploring prognostic markers and potential therapeutic targets for TNBC. For example, transcriptomic and proteomic analyses can identify key molecular features associated with TNBC aggressiveness and treatment response (131). Based on these data, predictive models can be developed to assess patient risk and treatment response. One study, using PANoptosis-related genes, successfully constructed a prognostic model containing four genes (BTN2A2, CACNA1H, PIGR, S100B). This model can not only predict TNBC patient prognosis but also recommend suitable therapies for different risk groups through immune infiltration and drug sensitivity analysis, embodying a complete pathway from risk assessment to personalized treatment guidance (132). Additionally, predictive models developed by integrating multi-gene mutation profiles (e.g., KRAS, PIK3CA, TP53) using machine learning algorithms can distinguish TNBC patients responding or not responding to first-line chemotherapy with high accuracy, providing powerful algorithmic tools for personalized therapy (133). This digital twin model can dynamically simulate tumor evolutionary trajectories under therapeutic pressure, predict the emergence of resistance, and assist clinicians in adjusting treatment plans. By combining empirical data from the PDO platform with simulation predictions from the digital twin model, the ultimate goal is to tailor optimal treatment sequences and combination strategies for each TNBC patient, truly transitioning from “one-size-fits-all” to “one-person-one-plan” precision medicine (134).

## 8 Challenges and future perspectives

### 8.1 Limitations and caveats

Several limitations of the current evidence should be acknowledged. First, much of the data on mast cell plasticity and metabolic regulation of immune cells derives from preclinical models, and direct evidence in human TNBC remains limited. Second, the phase 2 trial of cromolyn sodium, while promising, was small and single-arm; larger randomized trials are needed to confirm efficacy. Third, many of the therapeutic strategies discussed in this review—including cytokine modulation, metabolic interventions, and cell-based therapies—are at early stages of investigation and have not been validated in clinical settings. Readers should interpret these potential avenues with appropriate caution.

### 8.2 Technical challenges

Constructing organoid models that can accurately simulate the immune microenvironment and therapy resistance of TNBC is a key technological platform for deeply deciphering the synergistic mechanisms between mast cell plasticity and metabolic reprogramming. However, current organoid models face multiple challenges at both construction and application levels. First, the long-term survival and functional maintenance of immune cells in organoids is a core challenge. Taking mast cells as an example, as tissue-resident immune cells, their survival, phenotype, and function are highly dependent on complex microenvironmental signals, including cytokines, growth factors, and cell-cell contacts (56). Existing culture systems often struggle to maintain these key signals long-term *in vitro*, leading to rapid inactivation or phenotypic drift of mast cells in organoids, failing to truly reflect their dynamic changes within the *in vivo* tumor microenvironment and their regulatory role in immunotherapy. Therefore, developing co-culture systems that simulate interactions with various stromal cells like cancer-associated fibroblasts, endothelial cells, and neurons, and optimizing cytokine combinations, are crucial for maintaining the function of immune cells like mast cells.

Current organoid models have inherent limitations in simulating complex physiological structures *in vivo*. The tumor’s vascular system and innervation play indispensable roles in regulating local metabolism, immune cell migration, and intercellular communication. For example, the abnormal structure of tumor vessels affects the delivery of nutrients and oxygen, directly shaping the metabolic reprogramming of cells including mast cells and tumor cells. Neural signals may affect immune cell function by releasing neurotransmitters. Existing organoid models mostly lack functional vascular networks and innervation, making it difficult for models to fully reproduce the metabolism-immune interactions driven by hypoxia, nutrient competition, and neural signals within the *in vivo* TME. This structural simplification may omit key microenvironmental factors affecting mast cell plasticity and its synergy with tumor metabolism, thereby limiting comprehensive deciphering of immunotherapy resistance mechanisms.

Standardization of organoid-immune cell co-culture systems is a prerequisite for ensuring research data reliability, reproducibility, and ultimate clinical translation. Significant differences exist among laboratories in organoid sources, culture matrices, immune cell isolation and expansion methods, co-culture ratios, and time points, making research results difficult to directly compare and integrate. For instance, when assessing the impact of mast cells on organoid growth or treatment response, standardized cell numbers, activation states, and functional evaluation criteria are lacking. Promoting the establishment of standardized operating procedures and quality control systems, including standardized characterization of organoid genetic background, immune cell composition, and functional status, will greatly enhance the rigor of research in this field. Additionally, combining new technologies like spatial transcriptomics, e.g., Smart-seq3D, can reveal the spatial distribution and gene expression relationships of cells within organoids, providing a finer dimension for standardized assessment (135). Only by overcoming these technical and model-level challenges can more realistic and stable research platforms be constructed, enabling in-depth exploration of the specific mechanisms of mast cells in TNBC immunotherapy resistance and laying a solid foundation for developing effective intervention strategies.

### 8.3 Biological and translational medicine challenges

The mechanisms of immunotherapy resistance in TNBC are complex. The synergistic role of mast cell plasticity and metabolic reprogramming provides a new perspective for understanding this challenge, but its biological and translational medical application still faces multiple challenges. The regulatory network governing mast cell plasticity is extremely complex, involving substantial redundancy and feedback mechanisms, making intervention strategies targeting single pathways potentially limited in effect or easily compensated. TNBC itself exhibits high tumor heterogeneity, which is not only reflected at the genomic level but also profoundly affects the functional state of immune cells in the tumor microenvironment (136). Single-cell multi-omics analysis reveals highly heterogeneous immune cell subsets in the TNBC TME. They interact through complex intercellular communication networks, collectively shaping an immunosuppressive environment (137). In this context, mast cells, as key regulators in the TME, have their phenotype and function finely regulated by multiple signaling pathways, including Wnt, Hedgehog, Notch, etc., which also play important roles in breast cancer stem cell (BCSC) maintenance and drug resistance. Therefore, attempting to stabilize or reprogram mast cell function through a single target (like a specific cytokine or receptor) may fail due to compensatory activation of signaling networks, highlighting the necessity of developing multi-target combination intervention strategies.

Intervention strategies targeting metabolic reprogramming, such as targeting glycolysis, show therapeutic potential, but their potential systemic side effects may severely limit clinical application. Metabolic intervention drugs often lack tumor selectivity and may cause toxicity to normal tissues (especially organs with high metabolic demands). Drugs targeting glutathione metabolism or iron homeostasis may cause widespread oxidative stress or iron overload. To overcome this obstacle, developing tumor-targeted delivery systems or more selective inhibitors is crucial. Nanoparticle (NP) drug delivery systems responsive to TME characteristics (like low pH, hypoxia, high glutathione concentration) provide promising solutions for precise delivery of metabolic modulatory drugs (138). These smart delivery systems can enhance drug accumulation at the tumor site while reducing off-target effects and systemic toxicity. Additionally, integrating multi-omics analyses (e.g., metabolomics, proteomics) helps identify TNBC subtype-specific metabolic vulnerabilities, enabling the design of more selective small molecule inhibitors.

Tumor evolution and adaptive resistance must be considered. TNBC, especially metastatic TNBC, dynamically evolves under therapeutic pressure, potentially evading apMC-based immune attacks by altering its metabolic state or recruiting different immunosuppressive cells. Future translational research needs to integrate longitudinal sample analyses to assess the stability of the apMC phenotype during treatment and explore optimized strategies combining it with existing ICIs or other targeted therapies to overcome intrinsic and acquired resistance.

### 8.4 Future research directions

Future research should focus on several promising frontiers:

Developing Advanced “Immune Organoids” or “Assembloids”: Constructing more advanced 3D models that integrate tumor cells with a fuller repertoire of TME components (CAFs, endothelial cells, diverse immune cell subsets) is crucial for systematically deciphering intercellular communication, the formation mechanisms of immunosuppressive environments, and evaluating combination therapy strategies. Such models are expected to become powerful platforms for high-throughput screening of novel immunotherapies (e.g., bispecific antibodies, antibody-drug conjugates) and assessing their potential to reverse immunosuppression, providing more precise preclinical evidence for overcoming TNBC resistance to ICIs.

Integrating Spatiotemporal Multi-Omics and Live-Cell Imaging: Combining microfluidics, high-resolution live imaging, single-cell transcriptomics, and metabolomics within organoid platforms to track and quantify the “metabolic dialogue” between mast cells and TNBC cells in real-time across spatial and temporal dimensions. This multi-technology integration approach helps clarify how metabolic reprogramming drives mast cell plasticity changes and how these changes feedback regulate tumor cell proliferation, invasion, and chemotherapy resistance, providing unprecedented detailed insights for discovering key metabolic intervention nodes.

Engineering Mast Cells for Cell Therapy: Modifying mast cells using gene editing techniques (e.g., CRISPR-Cas9) to stably express anti-tumor active phenotypes and specifically target tumor sites is an innovative therapeutic idea. Furthermore, by loading engineered mast cells with chimeric antigen receptors (CAR) targeting tumor-associated antigens, they can be endowed with the ability to specifically recognize and kill TNBC cells, similar to the principles of CAR-T or CAR-macrophage (CAR-M) therapies. Such “living cell drugs” are expected to directly reshape the local immune microenvironment, overcome TME-mediated immunosuppression, and produce synergistic effects with existing ICIs, chemotherapy, or radiotherapy, providing entirely new treatment options for refractory TNBC patients unresponsive to current therapies.

Leveraging Artificial Intelligence (AI): Applying AI to integrate a multimodal data fusion framework that integrates organoid models with clinical cohorts—structurally aligning organoid drug response profiles, single-cell multi-omics (e.g., mast cell metabolic flux and epigenetic plasticity markers), high-content imaging features, and longitudinal patient clinicopathological information. By employing deep learning algorithms (such as graph neural networks and variational autoencoders), implicit associations between mast cell plasticity states and metabolic reprogramming nodes within high-dimensional data can be uncovered, enabling the development of digital tumor twins with spatiotemporal evolution capabilities.

Overcoming immunotherapy resistance in TNBC is a quintessential systems biology and engineering challenge. It demands continued collaboration across disciplines—oncology, immunology, metabolism, bioengineering, and data science. By deepening our understanding of the tumor ecosystem through advanced models like organoids and creatively designing multidimensional, spatiotemporally precise interventions, the goal of transforming TNBC into a manageable or even curable disease becomes increasingly attainable.
